# Transplacental sirolimus: a new treatment strategy for life-threatening fetal cardiac rhabdomyomas—a case report

**DOI:** 10.1186/s13023-025-03780-7

**Published:** 2025-06-09

**Authors:** Kaname Uno, Yoji Nomura, Masahiro Kawaguchi, Anna Ebina, Rina Imanishi, Satoru Kawai, Hiromi Hayakawa

**Affiliations:** 1 Department of Obstetrics, Aichi Children’s Health and Medical Center, 426-7, Morioka-cho, Obu, Aichi 474-8710 Japan; 2 Department of Obstetrics and Gynecology, Nagoya University Graduate School of Medicine, 65, Tsrumai-cho, Showa, Nagoya Japan; 3 Division of Oncology, Department of Clinical Sciences, Lund University, Medicon Village, Lund, Sweden; 4 Department of Pediatric Cardiology, Aichi Children’s Health and Medical Center, 426-7, Morioka-cho, Obu, Aichi Japan; 5 Department of Pediatric Neurology, Aichi Children’s Health and Medical Center, 426-7, Morioka-cho, Obu, Aichi Japan; 6 Department of Neonatal Intensive Care Unit, Aichi Children’s Health and Medical Center, 426-7, Morioka-cho, Obu, Aichi Japan

**Keywords:** Fetal cardiac rhabdomyoma, Tuberous sclerosis complex, MTOR inhibitor, Sirolimus, Transplacental treatment, Hypo left hemodynamic status, Subependymal giant cell astrocytoma

## Abstract

Large cardiac rhabdomyomas can disturb hemodynamic flow. Tuberous sclerosis complex is the most common cause of cardiac rhabdomyoma in fetuses. Recently, a mammalian target of rapamycin (mTOR) inhibitor effectively treated rhabdomyomas associated with tuberous sclerosis. Here, we report the effectiveness of an mTOR inhibitor in treating a fetus with large rhabdomyomas exhibiting severe heart failure when administered transplacentally. A 30-year-old pregnant woman was transferred to our hospital due to the presence of a cardiac tumor in the left ventricle (LV) of the fetus, which gradually enlarged to > 40 mm in diameter. The diastolic and systolic functions of the LV were completely disrupted. At 32 weeks of gestation, the fetus showed severe heart failure (cardiovascular profile score 7), with a high risk of death. The hemodynamic flow was consistent with hypoplastic left heart syndrome. Fetal magnetic resonance imaging revealed several cranial regions with 7.9 × 6.9 mm subependymal giant cell astrocytoma (SEGA). The fetus was clinically diagnosed with tuberous sclerosis, and therapy was initiated with maternally administered sirolimus, an mTOR inhibitor. Sirolimus effectively reduced the size of the tumor and improved the hemodynamics of the fetus. No severe complications occurred in the mother or fetus. The baby was born at 39 weeks of gestation with a mildly reduced LV ejection fraction. In addition, the dimensions of the intracranial SEGA decreased somewhat following initiation of maternal sirolimus treatment. Postnatal genetic testing confirmed a mutation in the *TSC2* gene. Currently, the baby is 3 months old with normal neurological development. Transplacental sirolimus administration can be a useful in treating large rhabdomyomas that disturb fetal hemodynamics.

**Supplementary Information:**

The online version contains supplementary material available at 10.1186/s13023-025-03780-7.

## Introduction

Fetal cardiac tumors are rare, with an incidence of approximately 0.13–0.23% [1–3]. Although tumors shrink spontaneously after birth, they can disrupt the fetal inflow or outflow tracts and lead to cardiac dysfunction and fetal demise [1, 2]. Rhabdomyoma is the most prevalent primary heart tumor in the pediatric age group (60–82%) [1, 2, 4]. According to observational studies on fetal cardiac tumors, approximately 25% of fetal patients with cardiac rhabdomyoma have a poor prognosis [1, 5]. Fetuses with congestive heart failure (cardiovascular profile score [CVPS] < 8) die in utero or before surgery [1, 6]. Until recently, no effective treatment strategies were established for these patients.

Tuberous sclerosis complex (TSC) is an autosomal dominant genetic disease [7, 8]. Mutations in the tumor suppressor genes *TSC1* and *TSC2,* which encode hamartin and tuberin, respectively, are responsible for the development of TSC. The hamartin–tuberin complex inhibits the mammalian target of rapamycin (mTOR) pathway, which plays a crucial role in the regulation of cellular growth and survival [9, 10]. Consequently, patients with TSC develop various benign tumors in several organs [10, 11]. Cardiac rhabdomyoma is the most commonly recognized feature of TSC in fetuses and neonates. Historically, surgical resection post-delivery has been the sole recourse for cardiac tumors that affect fetal hemodynamics [12, 13], but debulking surgery coupled with a premature delivery involves significant risk [1]. mTOR inhibitors are promising molecular agents for the treatment of TSC. Several studies have revealed beneficial effects of mTOR inhibitors on reducing the size of cardiac rhabdomyomas and subependymal giant cell astrocytomas (SEGA) in postnatal patients with TSC [14–16]. Their effectiveness and safety when administrated to the fetus transplacentally, however, remain controversial.

## Case report

**Fig. 1:**
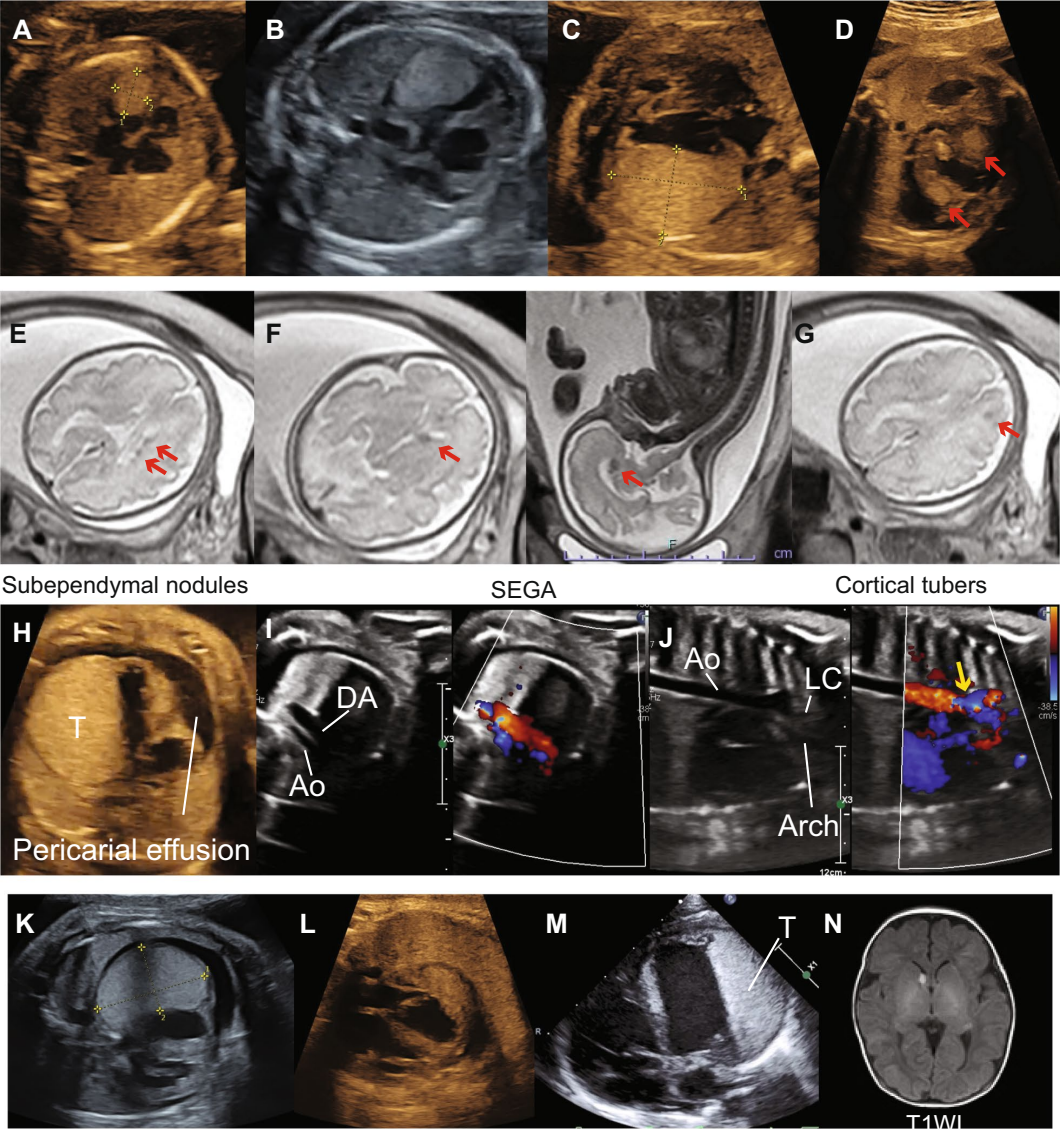
Changes in the size of cardiac rhabdomyomas in the fetus and after birth. **A**–**C** The tumor showed a gradual increase in size. The size increased to 15.6 × 12.9, 24.1 × 19.1, and 28.8 × 19.9 mm at **A** 24, **B** 26, and **C** 28 weeks of gestation, respectively. **D** In addition to the large cardiac tumor, multiple cardiac tumors (red arrows) were found at the septum and appendix. **E**–**G** Fetal MRI at 31 weeks of gestation revealed three characteristic cranial tumors: **E** subependymal nodules (horizontal section), **F** SEGA (horizontal and sagittal section), and **G** cortical tubers (horizonal section). The size of SEGA was 7.9 mm in diameter. **H** Cardiothoracic ratio > 50% with large pericardiac effusion. T indicates tumor lesion. **I** Decreased LV function similar to that of hypoplastic left heart. Transverse flow in the aorta compared to the flow in the ductus arteriosus. Aberrant flow in the aortic arch. **J** Blood flow for three common carotid arteries were supplied from the right ventricle through the ductus arteriosus (at 33 weeks of gestation before sirolimus treatment). **K** Cardiac tumor after two weeks of maternal sirolimus treatment. The tumor size decreased to 37.2 × 22.8 mm at 35 weeks of gestation. **L** The cardiac tumor was significantly decreased after five weeks of sirolimus treatment. The size of the tumor decreased to 26.2 × 13 mm, without pericardial effusion. The cardiovascular profile score (CVPS) improved from 7 to 9. **M** Cardiac echocardiography on postnatal day 0. The largest tumor was 36 mm in the LV lateral wall. The LV could bear the systemic circulation. **N** Neonatal MRI at postnatal day 14 showing SEGA. The size of the SEGA was 6.1 mm in diameter, which was smaller than that in the fetal MRI at 31 weeks of gestation (7.9 mm in diameter). MRI, magnetic resonance imaging; SEGA, subependymal giant cell astrocytoma; LV, left ventricle; T, rhabdomyoma tumor; DA, ductus arteriosus; Ao, aorta; LC, left common carotid artery

A 30-year-old pregnant woman (gravida 2, para 1) was referred to our hospital due to a suspected fetal cardiac tumor. She had no relevant medical, obstetrical, or family history. At 20 weeks of gestation, a small cardiac mass was suspected; subsequently, she was referred to our hospital. Fetal echocardiography revealed a single 9.4 × 7.2 mm round, homogeneous, and hyperechogenic cardiac mass on the left ventricle (LV) (Fig. 1A). No additional abnormal findings were noted.

The tumor, checked every 2 weeks, showed a gradual increase in size. The size increased to 15.6 × 12.9, 24.1 × 19.1, and 28.8 × 19.9 mm at 24, 26, and 28 weeks of gestation, respectively (Fig. 1B, C). The amniotic fluid index (AFI) also increased, mainly because the hard mass obstructed the esophagus (Supplementary Fig. 1 A). Moreover, at least three other cardiac tumors were found in the septum and appendix (Fig. 1D and Supplementary Movie 1). Fetal T2-weighted magnetic resonance imaging (MRI) at 31 weeks revealed several subependymal nodules, SEGA, and cortical tubers (Fig. 1E–G). The SEGA was 7.9 mm in diameter. Based on these findings, the fetus was clinically diagnosed with TSC.

The patient was hospitalized at 31 weeks of gestation due to threatened premature labor. The tumor further grew to 38.3 × 32.7 mm, and both inflow and outflow in the LV were inhibited with a 9.2 mm pericardiac effusion (Fig. 1H). The cardiothoracic area ratio was > 50%, without any arrhythmia. The flow of the foramen ovale was left to right due to diastolic dysfunction of the LV (Supplementary Fig. 1B). The cardiovascular profile score (CVPS) was 7, suggesting severe fetal heart failure. Following discussions with multidisciplinary teams, debulking surgery for iatrogenic premature delivery was considered high risk; instead, maternal sirolimus administration, which could be effective transplacentally in improving fetal hemodynamics, was considered. Written informed consent was obtained from the patient and her husband. At 33 weeks the tumor had enlarged to 43.5 × 36.5 mm, with a 10.9 mm large pericardiac effusion. LV fractional shortening was 0.078. The flow in the ascending aorta was reversed from the ductus arteriosus, consistent with a hypo left hemodynamic status (Fig. 1I, J, Supplementary Fig. 1 C–F, and Supplementary Movie 2).

**Fig. 2:**
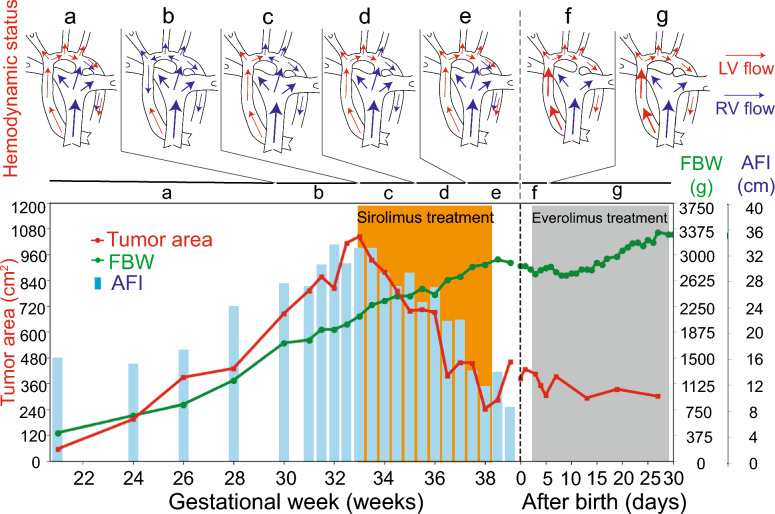
Changes in tumor area, FBW, and AFI in the clinical course before, during, and after sirolimus treatment. The changes in hemodynamic status are presented on topmost part of the figure. Fetal cardiac hemodynamic flow significantly improved with sirolimus treatment, whereas the LV function was disturbed with the huge cardiac mass. Small letters **a**–**g** represent the hemodynamic status in each period. Each letter represents at **a** 20–29, **b** 30–33, **c** 34–35, **d** 36–37, and **e** 38–39 weeks of gestation. **f** and **g** represent postnatal day 0–5, and 6–30, respectively. The tumor significantly increased until the start of sirolimus treatment and then subsequently shrank. The estimated fetal body weight had increased regardless of sirolimus initiation. The AFI fell within the normal range after sirolimus treatment. FBW, fetal body weight; AFI, amniotic fluid index; LV, left ventricle

After considering the potential maternal risks with unknown fetal benefits and obtaining approval for the use of sirolimus from the ethics committee of our hospital, oral maternal sirolimus treatment was initiated at 4 mg/day from 33 + 3 weeks of gestation with target sirolimus trough level at 5–12 ng/mL. There were no severe side effects except for mild oral stomatitis. In the second week, the tumor size decreased to 37.2 × 22.8 mm (Fig. 1K), and the LV output improved. At 37 weeks of gestation, the size of the tumor decreased to 26.2 × 13 mm. The flow from the LV reached the ductus arteriosus without pericardial effusion, and the AFI was within the normal range. The CVPS improved from 7 to 9 (Fig. 1L). We discontinued the transplacental sirolimus treatment at 38 weeks of gestation because of concerns regarding impaired wound healing. Figure 2 presents a summary of the clinical course of this case, and supplementary table shows the maternal laboratory data before, during, and after sirolimus treatment.

At 39 weeks, the patient underwent induction of labor due to the risk of tumor enlargement after discontinuing sirolimus treatment. She subsequently gave birth by cesarean section due to failure to progress over 2 days. The newborn female baby was delivered at 39 + 2 weeks of gestation (weight, 2,831 g; Apgar scores 8 and 9 at 1 and 5 min, respectively). The sirolimus levels in the umbilical artery and maternal blood were below sensitivity (< 1.0 ng/mL) at the time of delivery. Histopathological examination of the placenta revealed normal findings, without invading immune cells, signs of inflammation, or blood clots. The maternal postoperative course was unremarkable. The LV ejection fraction of the neonate was 52%, which was mildly decreased (Fig. 1M). No hypopigmentation patches were observed. Everolimus, another mTOR inhibitor, was initiated on day 2. MRI at postnatal day 14 revealed several subependymal nodules, SEGA, and cortical tubers. Interestingly, the size of SEGA was slightly decreased compared with that at 31 weeks of gestation (6.1 vs. 7.9 mm) (Fig. 1N and Supplementary Fig. 1G). Postnatal genetic testing confirmed a heterozygous mutation in *TSC2* (NM_000548.5, c.220G>T, p.Glu74 Ter). This mutation is classified as a pathogenic mutation in the ClinVar database (https://www.ncbi.nlm.nih.gov/clinvar/) [17]. The cardiac tumor size had decreased to 25 × 12 mm without any hemodynamic disturbances by postnatal day 30 (Supplementary Fig. 1H). The baby is currently 3 months old with normal neurological development. We will continue to observe the patient’s neurological development considering the in utero initiation of mTOR inhibitor.

## Discussion

Large cardiac rhabdomyomas diagnosed in fetuses by ultrasound or MRI may be reasonably presumed to be secondary to tuberous sclerosis complex (TSC). Transplacental treatment via maternal sirolimus administration may reduce the size of tumors and improve the fetal hemodynamic status pre-delivery as first reported by Barnes et al. in 2018 [18]. The risk of adverse symptoms might be ameliorated when the target maternal sirolimus trough level is set at 5–12 ng/mL owing to its higher level incord blood. Therefore, transplacental sirolimus administration in fetuses with TSC exhibiting life-threatening cardiac conditions appears to be a suitable treatment to save lives, with mild adverse effects.

While the incidence of primary heart tumors in infants is rare [1–3], unexpected deaths related to heart failure and cardiac arrhythmia do occur. Two observational studies with fetal cardiac tumors revealed a significantly higher risk of poor prognosis when tumors were > 20 mm or when fetuses showed heart failure [1, 5]. In our case, the tumor was very large (> 40 mm in diameter) and caused severe heart failure. Sirolimus treatment in utero reduced the tumor size and, moreover, allowed for extended gestational weeks, as prematurity is a factor with a worse prognosis [1].

A definitive diagnosis of TSC in utero can be challenging [7]. Several studies have confirmed mutations in *TSC1* or *TSC2* through amniocentsis before initiating mTOR inhibitor therapy [18–20]; however, the diagnosis of TSC in utero is, in fact, not difficult when the appropriate modalities are used and clinicians have a high index of suspicion. Amniocentesis is an invasive procedure that patients may choose to decline [21], and 10–25% of patients with TSC do not have mutations in *TSC1* or *TSC2* [4, 11, 22]. There is almost never a need for invasive genetic testing to establish the diagnosis of TSC in a fetus. Normally, diagnosis with TSC requires the presence of at least two major clinical features [10, 11]. Among them, only cardiac rhabdomyomas and brain tumors may be found during fetal development. Fetal brain MRI has effectively revealed the presence of subependymal nodules in approximately 60% of cases [3, 7, 23, 24]. In our case, MRI clearly revealed subependymal nodules and SEGA at 31 weeks of gestation. We believe that echocardiography and fetal MRI are reliable tools in utero to clinically diagnose TSC, as was the fetus in this case, to initiate sirolimus treatment without prenatal diagnostic genetic testing.

Accumulating evidence has demonstrated the efficacy of mTOR inhibitors in neonatal patients with TSC having cardiac rhabdomyomas [9, 13, 14, 20, 25]. To date, six studies (eight cases) have reported the use and efficacy of sirolimus in treating fetal cardiac rhabdomyomas with clinical manifestations without any severe side effect for pregnant women [18, 19, 23, 26–28]. However, mTOR inhibitors can cause several severe side effects, including immunosuppression and pneumonitis [9, 13]. Transplacental sirolimus treatment can be harmful to mothers without TSC symptoms, while maternal exposure is limited in a few months. Some reports have shown 132–163% higher levels of sirolimus in cord blood than in maternal serum [29, 30]. In this study, we set the target serum sirolimus level at 5–12 ng/mL, whereas previous studies used 10–15 ng/mL [18, 19]. Fetal growth restriction was reported during sirolimus treatment in two cases [19, 23]. In this study, the estimated fetal body weight was within the normal range even after treatment with sirolimus.

To our knowledge, this is the first report to set lower maternal sirolimus levels based on previously reported cord blood concentrations. Moreover, we highlight the potential benefits of sirolimus treatment for SEGA and for improving AFI to reduce the risk of premature delivery. This study also demonstrates current limitations and caveats associated with such cases. A thorough assessment of the fetal brain using ultrasonography alone is difficult. The differences between everolimus and sirolimus are unclear in terms of decreasing tumor size for cardiac rhabdomyomas and SEGA. To determine the effectiveness of transplacental sirolimus treatment, further case studies are necessary to reduce the risk of publication bias.

## Supplementary Information

Supplementary file 1: Supplementary Figure The amniotic fluid index at 30 weeks of gestation. AFI was > 25 after 20 weeks of gestation, mainly because the hard mass obstructed the esophagus and caused dysphagia. The flow of the foramen ovale was left-to-right due to diastolic dysfunction of the LV. LA; left atrium, RA; right atrium, RV; right ventricle, FO; foramen ovale, T; tumor lesion. Left or right systolic function assessed by fractional shortening. LVFS improved after sirolimus treatments. Z-score of RVFS and LVFS.The trend of velocity Time Integralof aorta and pulmonary artery. The changes of cortico throracic area ratio with or without pericardiac effusion. p.e; pericardiac effusion. The sub ependymal giant cell astro cytomaat postnatal day 14. The tumor diameter was 6.1 mm.. Echocardiography at the time of discharge. The cardiac tumor size had decreased to 25 × 12 mm without any cardiac disturbances at postnatal day 30

Supplementary file 2.

Supplementary file 3.

Supplementary file 4.

## Data Availability

Not applicable.

## Abbreviations

mTOR: Mammalian target of rapamycin
LV: The left ventricle
CVPS: Cardiovascular profile score
TSC: Tuberous sclerosis complex
SEGA: Subependymal giant cell astrocytoma
AFI: Amniotic fluid index
MRI: Magnetic resonance imaging

## References

[1] Niewiadomska-Jarosik K, Stańczyk J, Janiak K, Jarosik P, Moll JJ, Zamojska J, et al. Prenatal diagnosis and follow-up of 23 cases of cardiac tumors. Prenat Diagn. 2010;30:882–7. 10.1002/pd.2586. 20715118 10.1002/pd.2586

[2] Becker AE. Primary heart tumors in the pediatric age group: a review of salient pathologic features relevant for clinicians. Pediatr Cardiol. 2000;21:317–23. 10.1007/s002460010071. 10865004 10.1007/s002460010071

[3] Sciacca P, Giacchi V, Mattia C, Greco F, Smilari P, Betta P, et al. Rhabdomyomas and tuberous sclerosis complex: our experience in 33 cases. BMC Cardiovasc Disord. 2014;14:1–11. 10.1186/1471-2261-14-66. 24884933 10.1186/1471-2261-14-66 PMC4039990

[4] Chen J, Wang J, Sun H, Gu X, Hao X, Fu Y, et al. Fetal cardiac tumor: echocardiography, clinical outcome and genetic analysis in 53 cases. Ultrasound Obstet Gynecol. 2019;54:103–9. 10.1002/uog.19108. 29877000 10.1002/uog.19108

[5] Chao AS, Chao A, Wang TH, Chang YC, Chang YL, Hsieh CC, et al. Outcome of antenatally diagnosed cardiac rhabdomyoma: case series and a meta-analysis. Ultrasound Obstet Gynecol. 2008;31:289–95. 10.1002/uog.5264. 18307215 10.1002/uog.5264

[6] De Rosa G, De Carolis MP, Pardeo M, Bersani I, Tempera A, De Nisco A, et al. Neonatal emergencies associated with cardiac rhabdomyomas: an 8-year experience. Fetal Diagn Ther. 2011;29:169–77. 10.1159/000320483. 21109725 10.1159/000320483

[7] Goergen SK, Fahey MC. Prenatal MR imaging phenotype of fetuses with tuberous sclerosis: an institutional case series and literature review. Am J Neuroradiol. 2022;43:633–8. 10.3174/ajnr.A7455. 35332020 10.3174/ajnr.A7455 PMC8993194

[8] Jones AC, Shyamsundar MM, Thomas MW, Maynard J, Idziaszczyk S, Tomkins S, Sampson JR, et al. Comprehensive mutation analysis of TSC1 and TSC2—and phenotypic correlations in 150 families with tuberous sclerosis. Am J Hum Genet. 1999;64:1305–15. 10.1086/302381. 10205261 10.1086/302381 PMC1377866

[9] Qaderi S, Javinani A, Blumenfeld YJ, Krispin E, Papanna R, Chervenak FA, et al. Mammalian target of rapamycin inhibitors: a new-possible approach for in-utero medication therapy. Prenat Diagn. 2024;44:88–98. 10.1002/pd.6492. 38177082 10.1002/pd.6492

[10] Henske EP, Józwiak S, Kingswood JC, Sampson JR, Thiele EA. Tuberous sclerosis complex. Nat Rev Dis Prim. 2016;2:16035–54. 10.1038/nrdp.2016.35. 27226234 10.1038/nrdp.2016.35

[11] Northrup H, Krueger DA, International Tuberous Sclerosis Complex Consensus Group. Tuberous sclerosis complex diagnostic criteria update: recommendations of the 2012 international tuberous sclerosis complex consensus conference. Pediatr Neurol. 2013;49:243–54. 10.1016/j.pediatrneurol.2013.08.001. 24053982 10.1016/j.pediatrneurol.2013.08.001 PMC4080684

[12] Bader RS, Chitayat D, Kelly E, Ryan G, Smallhorn JF, Toi A, et al. Fetal rhabdomyoma: prenatal diagnosis, clinical outcome, and incidence of associated tuberous sclerosis complex. J Pediatr. 2003;143:620–4. 10.1067/S0022-3476(03)00494-3. 14615733 10.1067/S0022-3476(03)00494-3

[13] Inoue S, Inuzuka R, Kakiuchi S, Sato A, Matsui H. Successful treatment with everolimus for severe heart failure with large cardiac rhabdomyomas. Pediatr Int. 2022;64:5–7. 10.1111/ped.15219. 10.1111/ped.15219 35912461

[14] Saffari A, Brösse I, Wiemer-Kruel A, Wilken B, Kreuzaler P, Hahn A, et al. Safety and efficacy of mTOR inhibitor treatment in patients with tuberous sclerosis complex under 2 years of age—a multicenter retrospective study. Orphanet J Rare Dis. 2019;14:1–13. 10.1186/s13023-019-1077-6. 31053163 10.1186/s13023-019-1077-6 PMC6500021

[15] Krueger DA, Wilfong AA, Holland-Bouley K, Anderson AE, Agricola K, Tudor C, et al. Everolimus treatment of refractory epilepsy in tuberous sclerosis complex. Ann Neurol. 2013;74:679–87. 10.1002/ana.23960. 23798472 10.1002/ana.23960

[16] French JA, Lawson JA, Yapici Z, Ikeda H, Polster T, Nabbout R, et al. Adjunctive everolimus therapy for treatment-resistant focal-onset seizures associated with tuberous sclerosis (EXIST-3): a phase 3, randomised, double-blind, placebo-controlled study. Lancet. 2016;388:2153–63. 10.1016/S0140-6736(16)31419-2. 27613521 10.1016/S0140-6736(16)31419-2

[17] Landrum MJ, Lee JM, Benson M, Brown G, Chao C, Chitipiralla S, et al. ClinVar: public archive of interpretations of clinically relevant variants. Nucleic Acids Res. 2016;44:D862–8. 10.1093/nar/gkv1222. 26582918 10.1093/nar/gkv1222 PMC4702865

[18] Barnes BT, Procaccini D, Crino J, Blakemore K, Sekar P, Sagaser KG, et al. Maternal sirolimus therapy for fetal cardiac rhabdomyomas. N Engl J Med. 2018;378:1844–5. 10.1056/nejmc1800352. 29742370 10.1056/NEJMc1800352 PMC6201692

[19] Pluym ID, Sklansky M, Wu JY, Afshar Y, Holliman K, Devore GR, et al. Fetal cardiac rhabdomyomas treated with maternal sirolimus. Prenat Diagn. 2020;40:358–64. 10.1002/pd.5613. 31742705 10.1002/pd.5613

[20] Maász A, Bodó T, Till Á, Molnár G, Masszi G, Labossa G, et al. Three-year follow-up after intrauterine mTOR inhibitor administration for fetus with TSC-associated rhabdomyoma. Int J Mol Sci. 2023;24:1–11. 10.3390/ijms241612886. 10.3390/ijms241612886 PMC10454323 37629066

[21] Salomon LJ, Sotiriadis A, Wulff CB, Odibo A, Akolekar R. Risk of miscarriage following amniocentesis or chorionic villus sampling: systematic review of literature and updated meta-analysis. Ultrasound Obstet Gynecol. 2019;54:442–51. 10.1002/uog.20353. 31124209 10.1002/uog.20353

[22] Dragoumi P, O’Callaghan F, Zafeiriou DI. Diagnosis of tuberous sclerosis complex in the fetus. Eur J Paediatr Neurol. 2018;22:1027–34. 10.1016/j.ejpn.2018.08.005. 30279084 10.1016/j.ejpn.2018.08.005

[23] Ebrahimi-Fakhari D, Stires G, Hahn E, Krueger D, Franz DN. Prenatal sirolimus treatment for rhabdomyomas in tuberous sclerosis. Pediatr Neurol. 2021;125:26–31. 10.1016/j.pediatrneurol.2021.09.014. 34624607 10.1016/j.pediatrneurol.2021.09.014

[24] Jin N, Wu Y, Meng Q, Luo Q. Prenatal diagnosis of tuberous sclerosis complex: echocardiography, cranial magnetic resonance, and genetic testing of 40 cases with fetal cardiac tumors. Heliyon. 2023;9:1–8. 10.1016/j.heliyon.2023.e16980. 10.1016/j.heliyon.2023.e16980 PMC10361035 37484232

[25] Patel C, Abraham S, Ferdman D. Rapid regression of prenatally identified intrapericardial giant rhabdomyomas with sirolimus. CASE (Phila, PA). 2018;2:258–61. 10.1016/j.case.2018.07.003. 10.1016/j.case.2018.07.003 PMC6302122 30582086

[26] Vachon-Marceau C, Guerra V, Jaeggi E, Chau V, Ryan G, Van Mieghem T. In-utero treatment of large symptomatic rhabdomyoma with sirolimus. Ultrasound Obstet Gynecol. 2019;53:420–1. 10.1002/uog.20196. 30549350 10.1002/uog.20196

[27] Will JC, Siedentopf N, Schmid O, Gruber TM, Henrich W, Hertzberg C, et al. Successful prenatal treatment of cardiac rhabdomyoma in a fetus with tuberous sclerosis. Pediatr Rep. 2023;15:245–53. 10.3390/pediatric15010020. 36976727 10.3390/pediatric15010020 PMC10059978

[28] Dagge A, Silva LA, Jorge S, Nogueira E, Rebelo M, Pinto L. Fetal tuberous sclerosis: sirolimus for the treatment of fetal rhabdomyoma. Fetal Pediatr Pathol. 2022;41:800–6. 10.1080/15513815.2021.1948646. 34281475 10.1080/15513815.2021.1948646

[29] Park H, Chang CS, Choi SJ, Oh S, Roh CR. Sirolimus therapy for fetal cardiac rhabdomyoma in a pregnant woman with tuberous sclerosis. Obstet Gynecol Sci. 2019;62:280–4. 10.5468/ogs.2019.62.4.280. 31338346 10.5468/ogs.2019.62.4.280 PMC6629989

[30] Rodriguez-Antona C, Savieo JL, Lauschke VM, Sangkuhl K, Drögemöller BI, Wang D, et al. PharmVar GeneFocus: CYP3A5. Clin Pharmacol Ther. 2022;112:1159–71. 10.1002/cpt.2563. 35202484 10.1002/cpt.2563 PMC9399309

