# Supplementary figures and images for: Transplacental sirolimus: a new treatment strategy for life-threatening fetal cardiac rhabdomyomas—a case report
Source: Orphanet J Rare Dis. 2025 Jun 9;20:291. doi: 10.1186/s13023-025-03780-7 (PMC12147371; doi:10.1186/s13023-025-03780-7)

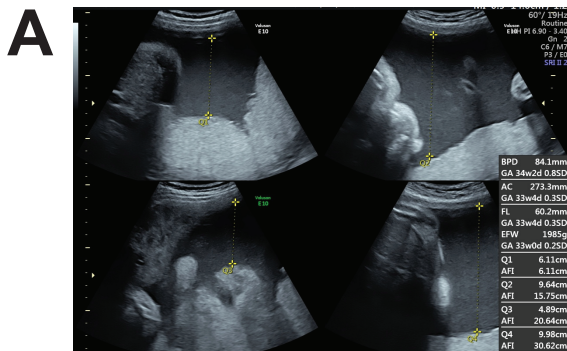

Amniotic fluid index

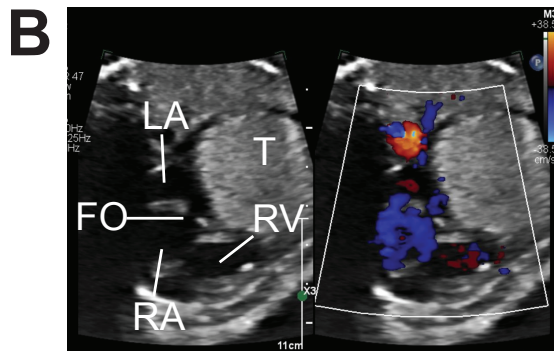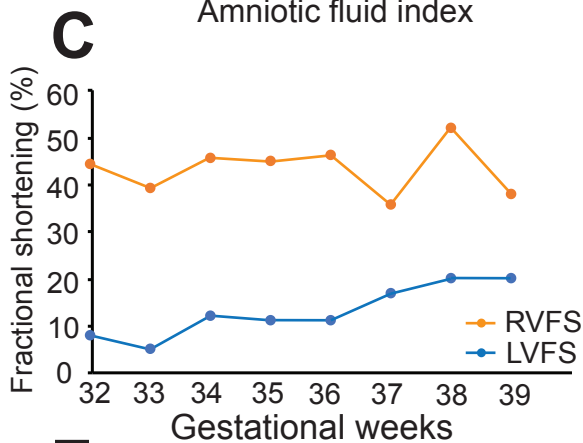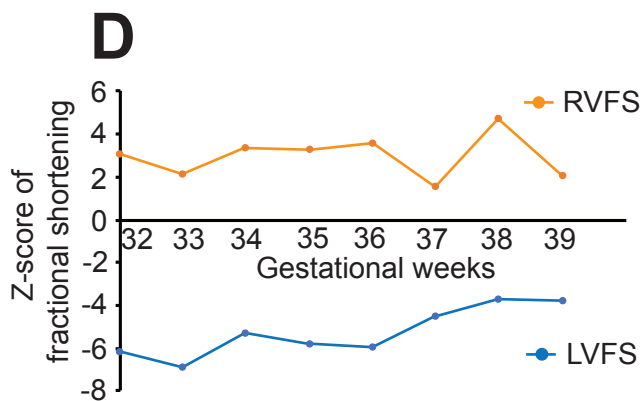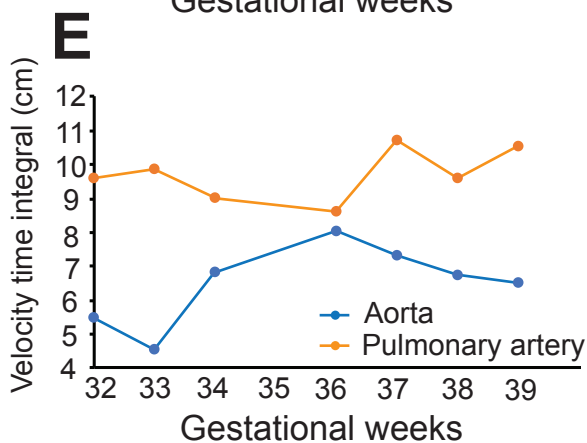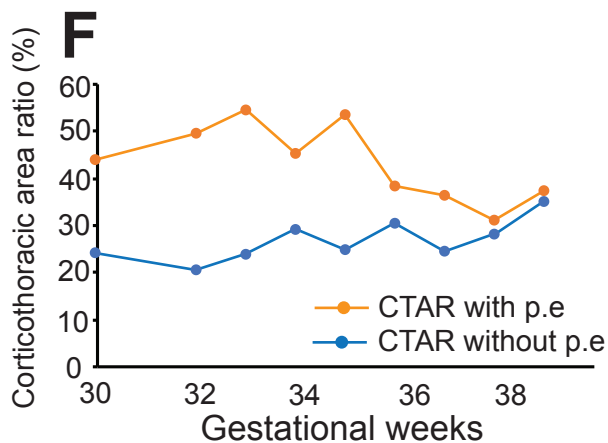

**G** Subependymal giant cell astrocytoma

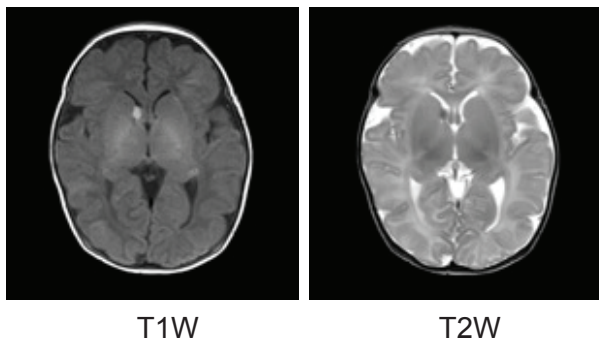

**H** Cardiac tumor at discharge

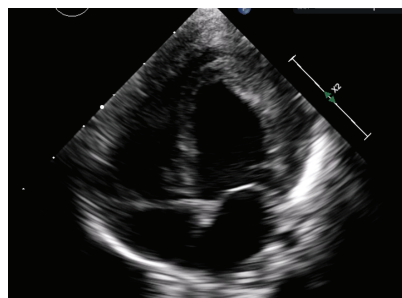

Supplement: Supplementary file 1 — Supplementary Figure The amniotic fluid index at 30 weeks of gestation. AFI was > 25 after 20 weeks of gestation, mainly because the hard mass obstructed the esophagus and caused dysphagia. The flow of the foramen ovale was left-to-right due to diastolic dysfunction of the LV. LA; left atrium, RA; right atrium, RV; right ventricle, FO; foramen ovale, T; tumor lesion. Left or right systolic function assessed by fractional shortening. LVFS improved after sirolimus treatments. Z-score of RVFS and LVFS.The trend of velocity Time Integralof aorta and pulmonary artery. The changes of cortico throracic area ratio with or without pericardiac effusion. p.e; pericardiac effusion. The sub ependymal giant cell astro cytomaat postnatal day 14. The tumor diameter was 6.1 mm.. Echocardiography at the time of discharge. The cardiac tumor size had decreased to 25 × 12 mm without any cardiac disturbances at postnatal day 30 [file 13023_2025_3780_MOESM1_ESM.pdf]
