# Supplementary material for: Transplacental sirolimus: a new treatment strategy for life-threatening fetal cardiac rhabdomyomas—a case report
Source: Orphanet J Rare Dis. 2025 Jun 9;20:291. doi: 10.1186/s13023-025-03780-7 (PMC12147371; doi:10.1186/s13023-025-03780-7)
Supplement: Supplementary file 2 [file 13023_2025_3780_MOESM2_ESM.docx]

| Supplementary Table. Laboratory data before, during, and after sirolimus treatment | | | | | | | | | | | |  |
| --- | --- | --- | --- | --- | --- | --- | --- | --- | --- | --- | --- | --- |
| **Characteristics** | | Before | During sirolimus treatment | | | | | After | After delivery | | |  |
| Weeks of Gestation wk | | 32 | 33 | 34 | 35 | 36 | 37 | 39 | day 1 | day 7 | day 30 |  |
| WBC | ul | 5930 | 6990 | 5880 | 5220 | 4500 | 5850 | 6320 | 6690 | 5370 | 5580 |  |
| Neutrophil | % | 74.2 | 65.7 | 64.8 | 67.6 | 60.5 | 66.6 | 68.9 | N/A | N/A | 54.1 |  |
| Lymphocyte | % | 19.4 | 27.3 | 28.6 | 25.9 | 31.1 | 26.5 | 25.8 | N/A | N/A | 40.1 |  |
| Hemoglobin | g/dl | 11.9 | 10.5 | 11.1 | 10.9 | 11.7 | 11.2 | 11.4 | 9 | 9.8 | 12.7 |  |
| Platelet | 10^4/ul | 28.1 | 26.7 | 26.3 | 26.1 | 26.8 | 27 | 28.9 | 26.9 | 40.9 | 31.5 |  |
| Total Protein | g/dl | 6.03 | N/A | 5.76 | 5.5 | 5.6 | 5.59 | N/A | 4.07 | 5.38 | N/A |  |
| Albumin | g/dl | 2.83 | N/A | 2.71 | 2.54 | 2.51 | 2.52 | N/A | 1.77 | 2.48 | N/A |  |
| BUN | mg/dl | 6.3 | 7.1 | 6.7 | 7.5 | 6.3 | 6 | 5.6 | 4.3 | 6.5 | 10.9 |  |
| Creatinine | mg/dl | 0.41 | 0.45 | 0.46 | 0.38 | 0.45 | 0.41 | 0.45 | 0.47 | 0.46 | 0.51 |  |
| AST | IU/l | 13 | 11 | 11 | 12 | 13 | 11 | 11 | 13 | 13 | 13 |  |
| ALT | IU/l | 10 | 8 | 7 | 6 | 6 | 6 | 5 | 4 | 10 | 11 |  |
| LDH | U/l | 144 | 147 | 136 | 139 | 167 | 153 | 172 | 193 | 150 | N/A |  |
| Triglyceride | mg/dl | 433 | N/A | 395 | N/A | N/A | 633 | N/A | 307 | 162 | N/A |  |
| Total-Cho | mg/dl | 293 | N/A | 288 | N/A | N/A | N/A | N/A | 222 | 253 | N/A |  |
| Low-Cho | mg/dl | 158 | N/A | 176 | N/A | N/A | 164 | N/A | N/A | N/A | N/A |  |
| Total bilirubin | mg/dl | 0.6 | N/A | N/A | N/A | N/A | 0.4 | N/A | 0.6 | 0.4 | N/A |  |
| PT-INR |  | 0.95 | N/A | N/A | N/A | N/A | 0.99 | N/A | N/A | N/A | N/A |  |
| Fibrinogen | mg/dl | 503 | N/A | N/A | N/A | N/A | 532 | N/A | N/A | N/A | N/A |  |
| D-dimer | ug/dl | 3.6 | N/A | N/A | N/A | N/A | 5.6 | N/A | N/A | N/A | N/A |  |
| CRP | mg/dl | 0.08 | N/A | 0.06 | N/A | N/A | 0.12 | N/A | N/A | 2.49 | N/A |  |
| WBC, white blood cell; BUN, blood urea nitrogen; AST, asparate aminotransferase; ALT, alanine aminotransferase; LDH, lactate dehydrogenase; Cho, cholesterol; PT-INR, prothrombin time-international normalized ratio; CRP, C-reactive protein. | | | | | | | | | | | |  |
|  |  |  |  |  |  |  |  |  |  |  |  |  |
